# J wave syndromes in patients with spinal and bulbar muscular atrophy

**DOI:** 10.1007/s00415-022-10992-5

**Published:** 2022-02-07

**Authors:** Karoline Steinmetz, Boris Rudic, Martin Borggrefe, Kathrin Müller, Reiner Siebert, Wolfgang Rottbauer, Albert Ludolph, Dominik Buckert, Angela Rosenbohm

**Affiliations:** 1 Department of Neurology, University of Ulm, Oberer Eselsberg 45, 89081 Ulm, Germany; 2 1st Department of Medicine, University Medical Centre Mannheim, Theodor-Kutzer-Ufer 1-3, 68167 Mannheim, Germany; 3 DZHK (German Centre for Cardiovascular Research), Partner Site Heidelberg/Mannheim, Mannheim, Germany; 4 Department of Cardiology, University of Ulm, Ulm, Germany; 5 Deutsches Zentrum Für Neurodegenerative Erkrankungen (DZNE), Partner Site Ulm, Ulm, Germany; 6 Institute of Human Genetics, University of Ulm and Ulm University Medical Center, Ulm, Germany

**Keywords:** Spinal and bulbar muscular atrophy, Brugada, J wave syndrome, Early repolarization, Cardiac magnet resonance imaging, Sudden cardiac death

## Abstract

**Background:**

Males with X-linked recessive spinobulbar muscular atrophy (SBMA) are reported to die suddenly and a Brugada electrocardiography (ECG) pattern may be present. A hallmark of this pattern is the presence of ST segment elevations in right precordial leads associated with an increased risk of sudden cardiac death.

**Objective:**

We aimed to detect subtle myocardial abnormalities using ECG and cardiovascular magnetic resonance imaging (CMR) in patients with SBMA.

**Methods:**

30 SBMA patients (55.7 ± 11.9 years) and 11 healthy male controls underwent 12-lead ECGs were recorded using conventional and modified chest leads. CMR included feature-tracking strain analysis, late gadolinium enhancement and native T1 and T2 mapping.

**Results:**

Testosterone levels were increased in 6/29 patients. Abnormal ECGs were recorded in 70%, consisting of a Brugada ECG pattern, early repolarization or fragmented QRS. Despite normal left ventricular ejection fraction (66 ± 5%), SBMA patients exhibited more often left ventricular hypertrophy as compared to controls (34.5% vs 20%). End-diastolic volumes were smaller in SBMA patients (left ventricular volume index 61.7 ± 14.7 ml/m^2^ vs. 79.1 ± 15.5 ml/m^2^; right ventricular volume index 64.4 ± 16.4 ml/m^2^ vs. 75.3 ± 17.5 ml/m^2^). Tissue characterization with T1-mapping revealed diffuse myocardial fibrosis in SBMA patients (73.9% vs. 9.1%, device-specific threshold for T1: 1030 ms).

**Conclusion:**

SBMA patients show abnormal ECGs and structural abnormalities, which may explain an increased risk of sudden death. These findings underline the importance of ECG screening, measurement of testosterone levels and potentially CMR imaging to assess cardiac risk factors.

## Introduction

Spinal and bulbar muscular atrophy (SBMA, MIM #313200), also known as Kennedy’s disease,) is an adult-onset lower motor neuron disorder with an X-linked inheritance pattern. SBMA is clinically characterized by slowly progressive weakness, atrophy and fasciculations of the bulbar and limb skeletal muscles evolving after the second decade. It is caused by an abnormal expansion of an unstable CAG trinucleotide repeat encoding a polyglutamin tract in the androgen receptor (*AR*) gene with >38 repeats producing the disease phenotype [1, 2]. In line with the chromosomal location of the *AR* gene in Xq12, SBMA follows an X-linked recessive inheritance pattern and, consequently, affects (almost) exclusively males.

The effect of the *AR* repeat expansion is androgen insensitivity with gynecomastia and decreased fertility and motor neuron degeneration which may lead to increased testosterone levels [3]. Metabolic abnormalities are frequently reported, especially type 2 diabetes and dyslipidemia [4, 5]. A progressive flaccid weakness leads to motor impairment in arms, legs and bulbar muscles and mortality is slightly increased (10-year survival of 82% compared to 95% among age-matched controls, mean age at diagnosis 54 years, *n* = 39) [6, 7]. Other typical symptoms are dysphagia, muscle cramps, tremor and elevation of creatine kinase due to bulbospinal degeneration of motor neurons and muscle involvement [4].

In 2014, 144 Japanese SBMA patients were examined to determine whether myocardial involvement was present [8]. From those, 48.6% had ECG abnormalities, most commonly ST-segment elevation in leads V1–V3. These right precordial abnormalities were attributed to Brugada syndrome in 17 of the 144 patients (11.8%). Two of these 17 patients were symptomatic and died suddenly. There were no statistically significant correlations between the Brugada ECG abnormalities and the *AR* gene CAG repeat length. No mutation in SCN5A, CACNA1C or CACNB2 gene associated with predisposition for Brugada syndrome could be detected in any of the 17 patients. In myocardial biopsies of 7 SBMA patients, a nuclear accumulation of the pathological androgen receptor protein could be detected, indicating myocardial involvement [8].

Brugada syndrome and early repolarization syndrome are well-known entities, being represented in epidemiological studies in 1–13% of the population [9–11]. Brugada syndrome (BrS) is predominantly diagnosed in males (80–90%) [12, 13]. In addition, the male gender is associated with an increased risk of life-threatening tachyarrhythmias in BrS.

Since there is a strong positive correlation between a high testosterone concentration and the prevalence of BrS, testosterone levels might also play a role in patients with SBMA.

The average testosterone serum concentration of SBMA patients is reported to be almost 100 ng/dl higher than controls. Most likely, these increased testosteron levels are due to the loss of sensitivity of the androgen receptor caused by the *AR* repeat expansion, thus leading to a compensatory increase in sex hormones (partial androgen insensitivity) [14] Despite high testosterone levels, patients suffer from testicular hypotrophy and reduced fertility as well as gynecomastia. Though testosterone levels are often increased in SBMA, they do not correlate with neuromuscular disease, such as onset and duration of paresis or CAG repeat length [15]. To corroborate the findings from Asia in an European cohort, we sought to study electrocardiographic phenotype as well as cardiac function and morphology in a cohort of European patients [8].

## Methods

Inclusion criteria were genetically confirmed diagnosis of SBMA and written consent to participate in the study. Exclusion criteria were the inability to provide detailed information on personal history, symptoms and co-medication. Patients opting to participate had to be able to undergo cardiac MRI examination. We recruited male SBMA patients from the outpatient clinic in the Department of Neurology, the University of Ulm (69 contacts, *n* = 16 had no interest in the study, *n* = 23 refused due to contrast allergy, motor deficits and long distance).

Thirty male patients with genetically confirmed SBMA entered the study. Information about individual disease history and characteristics, comorbidities, medication, neurological, and cardiac symptoms were obtained in structured personal interviews; in addition, blood samples were drawn. Since fever is known to unmask Brugada ECG changes due to temperature sensitivity of the sodium channels, a questionnaire included the history of febrile seizures, syncope, family history of sudden cardiac death, but also recorded the onset of muscle weakness and muscle cramps, body mass index (BMI), myalgia, comorbidities, comedications, dysphagia, dysarthria, sensory deficits, gynecomastia, respiratory symptoms and concomitant morbidities. Laboratory parameters were considered abnormal if they fell outside the established reference range. Disease onset was defined as the age of first symptom (gynecomastia or any muscular disturbances). Of the 30 patients entering the study, 29 gave informed consent for genetic testing of Brugada Syndrome-associated genes whereas one patient refused.

Ethical approval of the Ulm University Hospital ethics board was obtained for the study and was conform with the declaration of Helsinki (protocol no. 222/15). Written informed consent was obtained from each participant.

### Healthy controls

As controls, a cohort of male subjects from the ongoing multicenter study for the assessment of age- and gender-specific reference values for cardiac imaging markers (USAGE) was implemented. Healthy subjects lacking any cardiac comorbidity underwent a comprehensive CMR examination and a 12-lead ECG. Male healthy subjects at a wide age range served as controls.

### Analysis of Brugada syndrome associated genes

Peripheral blood-derived DNA was analyzed using Next Generation Sequencing after Targeted Enrichment (TruSight Rapid Capture Kit). Sequencing was conducted using the NextSeq High Output Kit v2.5 (300 cycles). The TruSight One® gene panel (Illumina) was applied for enrichment with bioinformatics filtering for genes associated with or linked to the Brugada syndrome (*ABCC9, CACNA1C, CACNA2D1, CACNB2, GPD1L, HCN4, KCND3, KCNE3, KCNH2, KCNJ8, PKP2, RANGRF, SCN10A, SCN1B, SCN2B, SCN3B, SCN5A, TRPM4*). Sequence data were mapped to the hg19 reference sequence. The threshold for minimal coverage was set to 20 and sequence analyses were limited to small variants in exonic regions of the named genes as well as in 10 bp of the adjacent introns. Using these criteria, a median of 97% of the target region has been covered.

### ECG

Standard and modified 12-lead-ECG were recorded and analyzed, precordial electrodes placed in the costal interspace 2–5 in modified ECGs. Brugada ECG pattern (BrP) was defined as coved or saddleback ST-segment elevation >2 mm (0.2 mV) in right precordial leads (V1–V3) [15]. Early repolarization was diagnosed when a J-point elevation >1 mm in >2 contiguous inferior and/or lateral leads was detected. ECGs were analyzed by two experienced cardiologists blinded to the medical history.

### Cardiac magnetic resonance imaging (CMR)

CMR was performed on a 1.5 T scanner (Achieva, Philips, Best, the Netherlands). The CMR protocol consisted of SSFP cine images in short and long-axis orientation, T1 mapping using a validated modified look locker inversion recovery (MOLLI) sequence in the 5(3)3 scheme, and a gradient spin echo sequence (GraSE) for T2 mapping. All CMR images were analyzed by two reviewers in consensus using a commercially available software (cvi42, Circle Cardiovascular Imaging Inc., Calgary, Alberta, Canada).

### Statistics

Statistical analyses were performed using GraphPad Prism 9 for Windows (GraphPad Software, Inc., La Jolla, CA, USA) and SAS, version 9.4 (SAS Institute, Chicago, IL, USA). Statistical significance was set at *p* < 0.05. Results from all tests were considered exploratory, in keeping with the study design and therefore, no adjustment for multiple testing was done.

## Results

A total of 30 male patients with genetically confirmed SBMA were included in the study. Mean age was 55.7 ± 11.9 years. Of the 30 patients, four reported previous syncopal episodes. Family history of sudden cardiac death was present in 7 patients and syncope in another 3 patients. None of them reported infantile febrile seizures. Cardiovascular diseases were noted in 16 patients (53%), most commonly arterial hypertension (*n* = 14). Atrial fibrillation was reported in three patients and a former myocardial infarction in another two patients. Five patients were previously diagnosed with type-2 diabetes.

The number of CAG repeats in the *AR* gene ranged from 40 to 67 repeats (median 47 repeats). All patients suffered from motor paresis (bulbar *n* = 23, spinal *n* = 30), and additionally, myalgias (*n* = 12), muscle cramps (*n* = 21), tremor (*n* = 29), sensory deficits (*n* = 19) and a history of laryngospasm (*n* = 21) were reported. Clinical characteristics and comedication in patients and controls are displayed in Table 1.

**Table 1:** Cardiovascular characteristics and comedication of SBMA patients and controls

	SBMA patients (*N* = 30)	Controls (*N* = 11)
Age (years), mean ± SD	55.7 ± 11.9	41.3 ± 14.0
Arterial hypertension (AHT) *n*, (%)	14 (46.7)	2 (18.2)
Diabetes, *n* (%)	5 (16.7)	0
ACE inhibitor or angiotensin-1 receptor antagonist, *n*	7	–
Beta-blocker, *n*	7	–
Other antihypertensive medication, *n*	6	–

*ACE* angiotensin converting enzyme, *SBMA* spinobulbar muscular atrophy

### Analyses of genes associated with Brugada syndrome

In none of the 29 patients with SBMA a pathogenic or likely pathogenic variant in the 18 genes analysed was detected. In three of the 29 patients, variants of unknown significance were detected.

### Blood biomarkers

Creatine kinase was elevated in 29/30 patients as well as CKMB (Table 2) Troponin T (TnT) was elevated in 22/25 patients (median 34 ng/l, IQR 17–44, normal <14 ng/l). NT-proBNP was elevated in 4/30 patients indicating heart failure. Hba1c was elevated in 4/29 patients, mean Hba1c levels were 5.5 ± 0.7%. Testosterone levels were elevated in 21% (*n* = 6) with a maximum value of 10.50 µg/l (reference range 1.93–7.40 µg/l), estradiol levels were also elevated in 21% (*n* = 6), but only 3 patients showed both levels elevated (see further details in Table 2). Androgen sensitivity index (product of luteinizing hormone and testosterone) was normal in all patients. Three of the patients with elevated testosterone levels had a family history of sudden cardiac death.

**Table 2:** Selected blood biomarkers in SBMA patient cohort

Blood Biomarker (reference range)	Mean ± std	Abnormal	Abnormal (%)
CK U/l (20–200)	1167 ± 1003	29/30 (↑)	96.7%
CK-MB µg/l (<6.22)	29.5 ± 24.5	24/25 (↑)	96.0%
Troponin T ng/l (<14)	40.16 ± 30.62	22/25 (↑)	88.0%
NT-proBNP pg/l (<85.5)	100.8 ± 302.4	4/25 (↑)	16.0%
Cholesterol mmol/l (<5.0)	5.75 ± 1.72	19/29 (↑)	65.5%
Triglycerides mmol/l (<1.7)	2.69 ± 3.30	16/29 (↑)	55.2%
HDL mmol/l (>1.0)	1.45 ± 0.40	6/28 (↓)	21.4%
LDL mmol/l (<3.0)	3.51 ± 0.98	20/28 (↑)	71.4%
17OH-Progesteron µg/l (0.05–1.60)	0.97 ± 0.64	4/28 (↑)	14.3%
Androstendion µg/l (0.7–3.6)	1.67 ± 0.80	3/28 (2↓, 1↑)	10.7%
DHEA-Sulfat µg/dl (44.3–3331.0)	246 ± 140	1/28 (↓)	3.6%
FSH IU/l (1.5–12.4)	7.53 ± 5.35	4/28 (↑)	14.3%
LH IU/l (1.70–8.60)	7.91 ± 3.02	8/28 (↑)	28.6%
Estradiol ng/l (11.3–43.2)	33.6 ± 13.2	6/28 (↑)	21.4%
Progesterone µg/l (<0.149)	0.25 ± 0.23	10/28 (↑)	35.7%
SHBG nmol/l (4.0–15.2)	60.3 ± 21.1	8/29 (↑)	27.6%
Testosterone µg/l (1.93–7.40)	5.59 ± 2.07	6/29 (↑)	20.7%
Androgen sensitivity index (ASI) (LH in IU/l × testosterone in nmol/l) (<138)	44.6 ± 25.9	0/28	0%

*CK* creatine kinase, *CK-MB* creatine kinase muscle-brain type, *DHEA* dehydroepiandrosterone, *FSH* follicle-stimulating hormone, *LH* luteinizing hormone, *SHBG* sex-hormone binding globulin

### ECG abnormalities

**Fig. 1:**
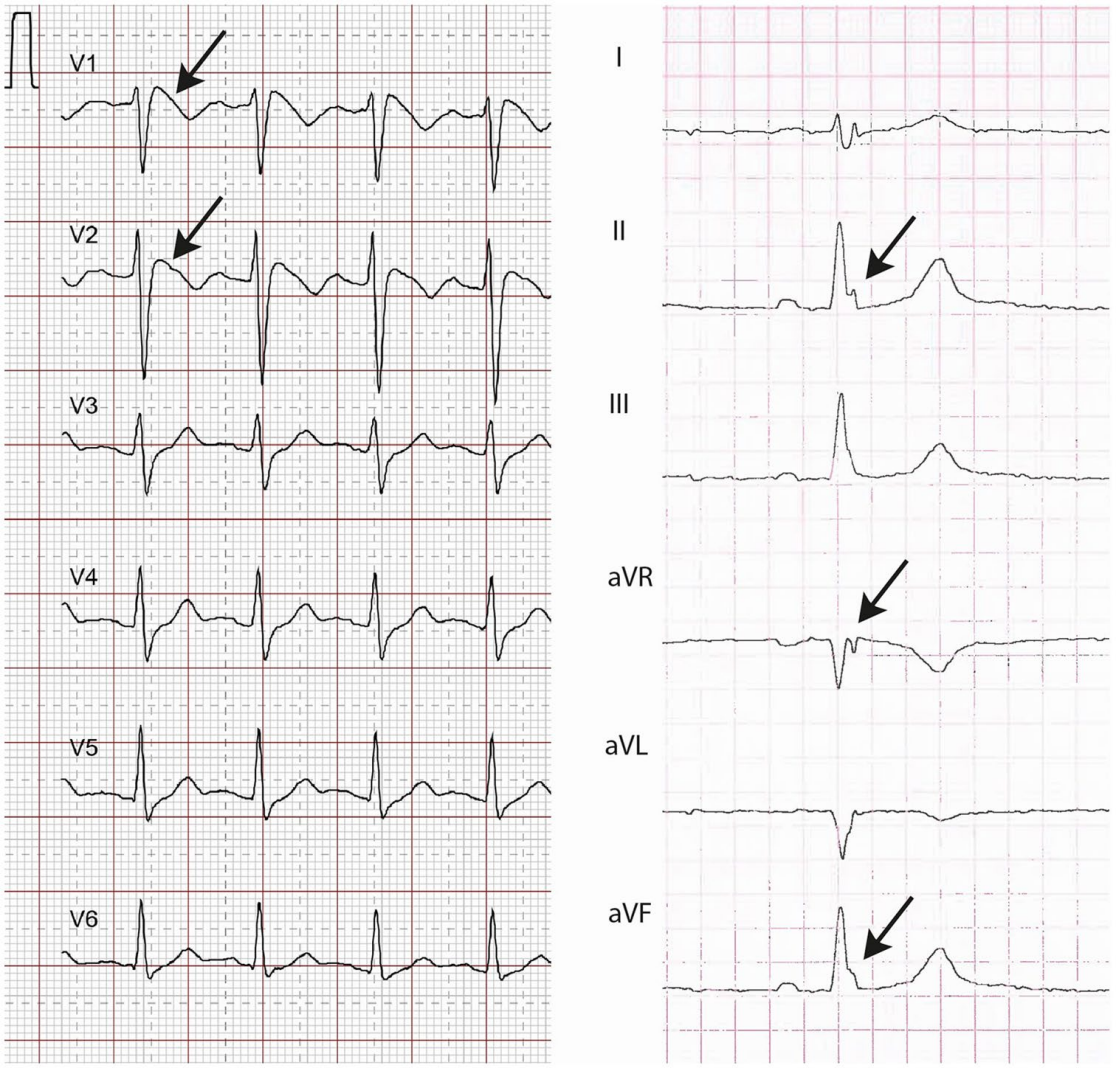
Representative examples of Brugada ECG pattern in V1–V6 (left side) and early repolarization pattern in II, aVR and aVF (right side)

Sinus rhythm was present in 28 and atrial fibrillation in another 2 patients. Left anterior hemiblock was present in 3 and incomplete right bundle block in another 3 patients. In 17 patients, an early repolarization pattern was noted. Two patients displayed a typical Brugada ECG pattern at rest (Table 3); these patients did not have variants of unknown significance in the genetic Brugada panel. BrP was observed in modified precordial leads. One patient with BrP additionally showed an early repolarization pattern. Representative examples are displayed in Fig. 1. Of the patients with elevated testosterone levels, ECGs showed early repolarization (*n* = 3) and a fragmented QRS complex (*n* = 1). CMR was normal in one of them whereas the remaining 2 had RV and LV strain abnormalities and also abnormal T1 values. The elevated cholesterol and LDL cases did not correlate with ECG abnormalities, of 19 elevated cholesterol levels, 10 displayed fragmented QRS complex. Otherwise, normal cholesterol levels displayed QRS fragmentation in 7/10. The Brugada ECGs had both increased cholesterol levels.

**Table 3:** ECG findings in SBMA patients and controls

ECG finding	SBMA patients		Controls	
Heart rate (bpm), mean ± std	72 ± 13		72 ± 15	
Atrial fibrillation, *n*	2/30		0/11	
Left anterior hemiblock, *n*	3/30		0/11	
Right bundle branch block, *n*	3/30		0/11	
Brugada ECG pattern, *n*	2/30		0/11	
Early repolarization pattern, *n*	17/30	Notching = 7	2/11	Notching = 1
		Slurring = 4		Slurring = 0
		Notching and slurring = 6		Notching and slurring = 1
Fragmented QRS complex, *n*	5/30		0/11	
QTc, ms	413 ± 24		418 ± 24	
Prolonged QTc, *n* (%)	2/30 (7)		0/11	
Pathological repolarization (Brugada or early repolarization or fQRS), *n* (%)	20/30 (67)		2/11 (18)	
Any pathological ECG, *n* (%)	21/30 (70)		2/11 (18)

### CMR findings

**Fig. 2:**
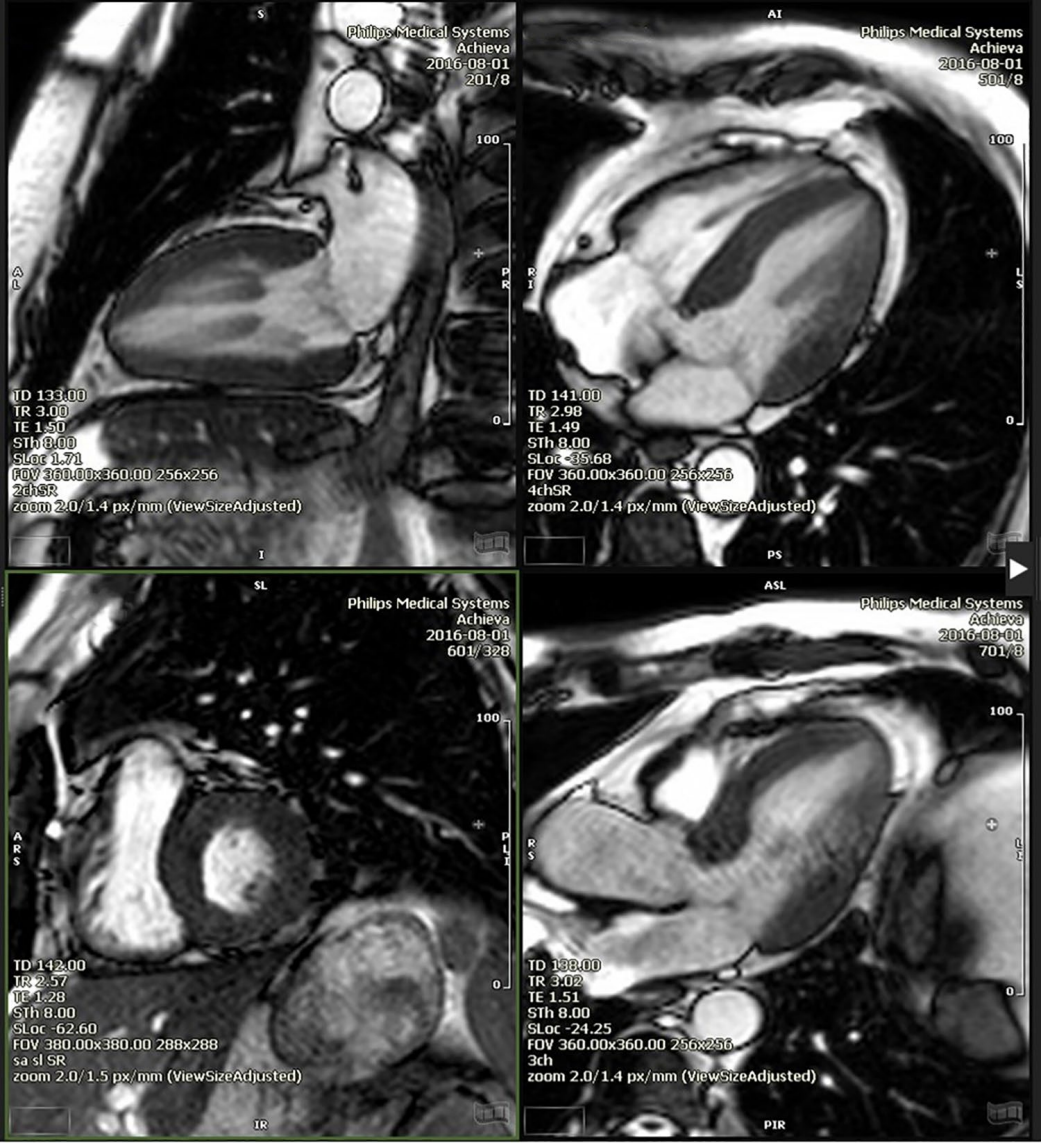
SMBA patient with concentric hypertrophy and prominent papillary muscles on cine-imaging. 2-chamber, 4-chamber, short axis and 3-chamber view

CMR was performed in 30 SBMA patients and 11 controls. The results are summarized in Table 4. Mean left and right ventricular ejection fraction (LVEF, RVEF) were in the normal range and did not differ from controls. Mean left ventricular ejection fraction was 66% (range 47–73%). End-diastolic volumes (left and right, displayed as indexes) differed from healthy controls and were lower in SBMA patients. Left ventricular hypertrophy was noted in 34% of SBMA patients (Fig. 2). Myocardial strain measures the degree of deformation of a myocardial segment from its initial length (end-diastolic) to its maximum length (end-systolic) and is expressed as a percentage. Right ventricular strain was not statistically different in SBMA patients compared to healthy controls (−23.8 ± 5.6% versus −20.7 ± 2.4%; *p* = 0.10). However, left ventricular strain was significantly higher in SBMA patients (−17.3 ± 3.2% versus −14.5 ± 1.3%; *p* = 0.01). LGE was not present in any patient. T2 mapping was pathological in 2/29 patients. Right ventricular strain did not differ in patients with or without repolarization abnormalities.

**Table 4:** CMR characteristics of patients and controls

	Unit	SBMA cohort, *n* = 30	Control cohort, *n* = 11	Unpaired *t*-test *p*-value
LVEF (%)	Mean ± SD	66.00 ± 4.95	64.40 ± 7.56	0.25
LVEDV index (ml/m^2^)	Mean ± SD	61.66 ± 14.67	79.10 ± 15.45	**0.0008**
RVEF (%)	Mean ± SD	64.13 ± 7.21	57.20 ± 6.11	**0.007**
RVEDV Index (ml/m^2^)	Mean ± SD	64.35 ± 16.42	75.30 ± 17.49	0.05
Hypertrophy *n*, (%)	Frequency	10/29 (34)	2/10 (20)	0.41
RV dilatation *n*, (%)	Frequency	1 (3)	0 (0)	0.56
T1 MAP nativ (ms)	Mean ± SD	1055 ± 51.5	992.9 ± 30.5	**0.001**
T1 path overall n, (%)	Frequency	17/23 (73.9)	1/10 (10)	**0.0004**
T2 path overall *n*, (%)	Frequency	2/29 (7)	0 (0)	0.41
LVEF < 55% *n*, (%)	Frequency	0 (0)	0 (0)	-
Presence of LGE *n*, (%)	Frequency	0	0	-
Any CMR abnormality and any repolarization disturbances in ECG (Brugada/J-wave/fQRS)	Frequency	14/25 (56)	-	-

CMR indexes related to body surface areas (BSA), which was calculated by the Dubois and Dubois regression formula BSA = 0.007184 × weight(Kg)^0.425^ × height[cm]^0.725^

*LVEF* left-ventricular ejection fraction, *LVEDVI* left-ventricular end-diastolic volume index, *RVEF* right-ventricular ejection fraction, *RVEDVI* right-ventricular end-diastolic volume index, *SBMA* spinobulbar muscular atrophy

**Fig. 3:**
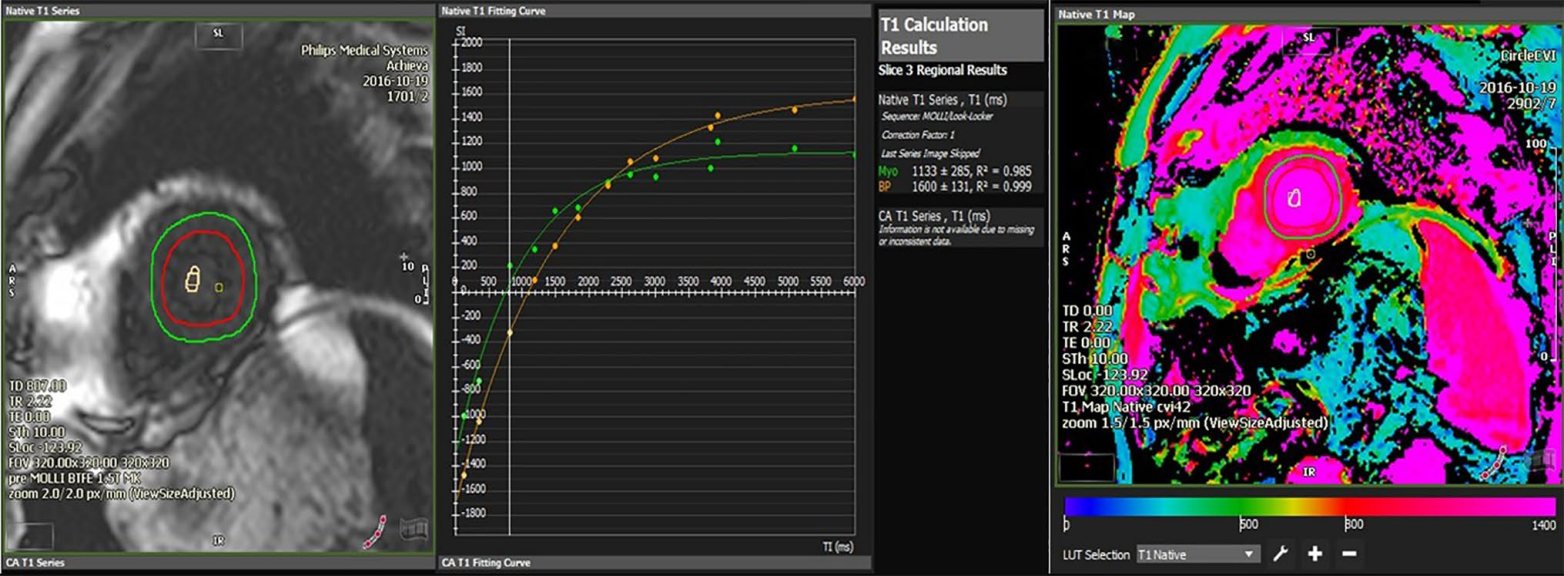
Native T1-mapping showing signs of increased diffuse fibrosis. Average T1 time 1133. ms on midventricular short axis MOLLI-sequence. Colour-coded look-up table on the right

Significantly higher global T1 values were measured in patients vs. controls (1055 ± 52 vs. 993 ± 31, *p* = 0.001) and cardiac T1 mapping was abnormal in 74% vs. 10% (*p* = 0.0004) (Fig. 3).

## Discussion

In the present study, a comprehensive cardiac evaluation of European SBMA patients was performed with standard and modified 12-lead ECG and CMR imaging. In a prior study of 25 SBMA patients from Scandinavia undergoing 12-channel ECG recording and echocardiography, there was no evidence of cardiomyopathy [16]. However, advanced imaging modalities, such as CMR, were not performed.

### ECG

In the present study, electrocardiographic abnormalities were found in two-thirds of patients with SBMA. These were mainly early repolarization pattern (17/30 patients), QRS fragmentation (5/30 patients) and two patients with a characteristic Brugada ECG pattern. Thus, we did not find the reported association between SBMA and BrS previously reported in a Japanese study to the same extent (6.6% BrP in the present study versus 11.8% in the Japanese cohort). However, provocation tests (e.g. ajmaline challenge) were not performed in the present study. The incidence of BrS as an autosomal dominant cardiac sodium channelopathy is reported to be higher in Southeast Asian populations [17], which may partially account for the reported association and higher prevalence in Japanese patients. Remarkably, we did not detect pathogenic or likely pathogenic variants in 18 genes associated with Brugada syndrome in the 29 patients in our cohort tested. Our findings indicate the presence of a large proportion of repolarization abnormalities (mainly early repolarization) as a frequent ECG pathology. This was not correlated to individual CMR findings.

J-wave syndromes include the BrS and the early repolarization syndrome. The occurrence of both entities is associated with an increased risk of life-threatening tachyarrhythmias, which may lead to sudden cardiac death [12, 18, 19]. Early repolarization patterns, either as “notching” or “slurring” of the terminal portion of the QRS are common in young and healthy individuals [20]. Three large studies concluded that the patterns of early repolarization in the inferior leads combined with a horizontal or lowered ST segment significantly increased the risk of sudden unexpected death [21–24].

In addition, we identified fragmentation of the QRS complex in 5 patients. A meta-analysis came to the conclusion that a fragmented QRS complex is associated with an increased risk of sudden cardiac death and overall mortality. However, fragmented QRS complexes can also be found in healthy controls [25, 26].

### ECG and steroid hormones

The testosterone-dependent nuclear accumulation of the androgen receptor plays a central role in the pathogenesis of SBMA. A study using the 5-alpha reductase inhibitor dutasteride inhibiting the conversion of testosterone to dihydrotestosterone, revealed autopsy-proven cardiac death opening a discussion on testosterone levels as a risk factor for cardiac complications [27]. BrS is often associated with increased testosterone levels [14], and testosterone is an important factor in the BrS ECG pattern among young patients [28]. Therefore, an association between subtle cardiac abnormalities and testosterone/steroid hormone levels was explored.

Six patients had elevated testosterone levels. Three of them had a family history of sudden cardiac death. The ECGs showed repolarization (*n* = 3) and a fragmented QRS complex (*n* = 1). CMR was normal in one of them whereas the remaining 2 had RV and LV strain abnormalities and also abnormal T1 values.

SBMA patients tend to have increased estradiol values due to aromatization of testosterone to estradiol in ineffective androgen receptor binding. Estradiol is also responsible for a QT-prolonging effect [29]. Furthermore, estradiol has proarrhythmogenic effects in patients with Long QT syndrome [30]. In contrast, progesterone decreases the occurrence of arrhythmias[30].

We also evaluated estradiol and progesterone levels in SBMA and analyzed them regarding potential prolongation of QT interval. 6 patients had increased estradiol values. The QT intervals were normal in 5 out of these 6 patients and only slightly prolonged in one patient (QTc = 460 ms). Increased progesterone values were observed in 10/30 patients. All of these patients had normal QTc values.

### CMR

Advanced CMR techniques are described to be a sensitive diagnostic tool to detect early morphological changes of the myocardium. Before left ventricular ejection fraction decreases, CMR findings can detect impaired right and left ventricular strain in acute myocarditis [31, 32]. Similar CMR abnormalities were reported in chemotherapy-induced toxic myocardiopathies [33].

Strain analysis seems to be more sensitive than ejection fraction measurements and conventional cardiac workup in muscular dystrophies (e.g. Duchenne dystrophy). LV circumferential strain and RV longitudinal strain were predictors of adverse arrhythmic outcome in a large population-based cohort of tetralogy of Fallot patients irrespective of LVEF [34, 35].

The observed combination of hypertrophy and increased left ventricular strain values points to hypercontractility of SBMA heart muscles. This may be interpreted as a minor form of diastolic heart failure, termed heart failure with preserved ejection fraction (HFpEF). Early observations in hypertrophic cardiomyopathies also show such hypercontractility and are often accompanied by myocardial fibrosis.

Native T1 in CMR is sensitive to intra- and extracellular free water content and is expected to be increased in edema, as can be seen in acute and chronic myocarditis [36]. Interstitial expansion is also increased in diffuse myocardial processes like fibrosis and can be abnormal before LGE is detected [37].

The cohort described here showed high frequencies of abnormalities in advanced CMR techniques with heart muscle hypertrophy, supraphysiological strain and elevated T1 measurement. Cardiac muscle abnormalities were also described in other motor neuron diseases. Heart muscle in amyotrophic lateral sclerosis (ALS) showed pathological T1 enhancement in 77% of the patients compared to 27% of controls and ejection volumes in the left and right heart were severely decreased in ALS patients [38].

The diffuse and rather subtle CMR changes described here seem to be features of the development of a seemingly non-severe cardiomyopathy with preserved systolic function. Our CMR findings are further supported by the elevated levels of cardiac troponin (TnT) and NTpro-BNP, which indicates subtle structural alternation, in the absence of clinical cardiomyopathy.

74% of SBMA patients showed T1 abnormalities and a combination of any repolarization abnormality with any CMR abnormality was seen in 56% of the cohort. 4/7 patients with positive family history for sudden cardiac death had a combination of pathological early repolarization in ECG and CMR strain or T1. Since these abnormalities were only seen with advanced CMR techniques, a combination of a positive family history concerning sudden cardiac death, ECG changes (early repolarization pattern) and strain analysis as well as T1 analysis in CMR seems to be a reasonable recommendation for the evaluation of cardiac risk factors in SBMA patients.

Concerning limitations, serial ECG recordings were not performed prospectively in all patients. Therefore, although unlikely, dynamic changes of electrocardiogram patterns may remain concealed. Also, specific provocation tests with sodium-channel blockers were not performed which could have further demasked potential underlying repolarization abnormalities in SBMA patients. Since the frequency of diabetes and arterial hypertension differed in cases and controls, some of the reported abnormalities in SBMA patients might be due to cardiovascular risk factors. Patients with metabolic heart disease can develop increased myocardial interstitial fibrosis as well as increased LV stiffness. Impaired systolic and diastolic function is described in arterial hypertension and diabetes [39, 40].

## Conclusions

The current findings further underline the importance of an initial CMR study in addition to ECG, since the highest frequency of pathological imaging was seen by the use of T1 CMR technique. In our study, hormonal status did not seem to correlate with the observed morphologic changes obtained by CMR, however sporadic fluctuations in testosterone levels may remain undetected but have an impact on the spontaneous occurrence of life-threatening tachyarrhythmias and sudden death.
